# Metal Homeostasis and Gas Exchange Dynamics in *Pisum sativum* L. Exposed to Cerium Oxide Nanoparticles

**DOI:** 10.3390/ijms21228497

**Published:** 2020-11-11

**Authors:** Elżbieta Skiba, Monika Pietrzak, Magdalena Gapińska, Wojciech M. Wolf

**Affiliations:** 1 Institute of General and Ecological Chemistry, Lodz University of Technology, Zeromskiego 116, 90-924 Lodz, Poland; monika.pietrzak@dokt.p.lodz.pl (M.P.); wojciech.wolf@p.lodz.pl (W.M.W.); 2 Laboratory of Microscopic Imaging and Specialized Biological Techniques, Faculty of Biology and Environmental Protection, University of Lodz, Banacha 12/16, 90-237 Lodz, Poland; magdalena.gapinska@biol.uni.lodz.pl

**Keywords:** cerium oxide nanoparticles, *Pisum sativum* L., environmental stress, hydroponic culture, metals uptake, photosynthesis

## Abstract

Cerium dioxide nanoparticles are pollutants of emerging concern. They are rarely immobilized in the environment. This study extends our work on *Pisum sativum* L. as a model plant, cultivated worldwide, and is well suited for investigating additive interactions induced by nanoceria. Hydroponic cultivation, which prompts accurate plant growth control and three levels of CeO_2_ supplementation, were applied, namely, 100, 200, and 500 mg (Ce)/L. Phytotoxicity was estimated by fresh weights and photosynthesis parameters. Additionally, Ce, Cu, Zn, Mn, Fe, Ca, and Mg contents were analyzed by high-resolution continuum source atomic absorption and inductively coupled plasma optical emission techniques. Analysis of variance has proved that CeO_2_ nanoparticles affected metals uptake. In the roots, it decreased for Cu, Zn, Mn, Fe, and Mg, while a reversed process was observed for Ca. The latter is absorbed more intensively, but translocation to above-ground parts is hampered. At the same time, nanoparticulate CeO_2_ reduced Cu, Zn, Mn, Fe, and Ca accumulation in pea shoots. The lowest Ce concentration boosted the photosynthesis rate, while the remaining treatments did not induce significant changes. Plant growth stimulation was observed only for the 100 mg/L. To our knowledge, this is the first study that demonstrates the effect of nanoceria on photosynthesis-related parameters in peas.

## 1. Introduction

Contemporary technology and science cannot advance without a parallel growth in nanotechnology and nanochemistry. Nowadays, nanomaterials are attracting continuously growing attention due to their unique properties. The latter originate from their dimensions, which extend the macroscale towards the atomic resolution [1,2,3,4]. Following highly appreciated The Nanodatabase as run by the Danish Ecological Council and the Danish Consumer Council, the number of products placed on the European market and containing nanoparticles has almost triplicated since 2012 [5,6].

Sooner or later, nanomaterials and their debris reach either water or soil environment [7,8,9]. This process is additionally prompted by intensive commercialization of engineered nanoparticles (ENPs), which are finding numerous applications in industry, agriculture, and medicine [10,11,12,13,14]. They are rarely immobilized in local environments and can migrate over relatively long distances [15,16,17]. The relevant data unanimously indicate that ENPs enter the environment at every stage of their life cycle, i.e., production, use, and waste disposal [18,19,20]. The final accumulation of ENPs creates novel environmental stress factors for living organisms. Regrettably, the resulting pattern is far from the simplicity, with complex additive interactions being rarely appreciated [21,22]. Obviously, further investigations in the subject are needed [23,24].

Cerium dioxide ENPs are important pollutants of emerging concern [25,26]. The natural content of Ce in soil depends on the location [27], with the worldwide average approaching 60 ppm [28]. Increasing production and consumption of nanoparticulate CeO_2_ make them one of the most widespread ENPs with significant participation in the global anthropopressure. Unfortunately, existing data on their impact on plant health and development are far from being consistent [29,30,31].

Plants are exposed to increasing concentrations of nanoparticles (NPs) in the environment [32,33,34]. This issue raises further questions on their bioaccumulation and trophic transfer in food products of plant origin [35,36]. The latter is associated with mechanisms of NPs toxicity as related to their size, shape, charge, and surface properties [37,38,39]. NPs present in either soil or soilless environments are absorbed by the plant root systems and further transported via the apoplastic or symplastic pathways. The former starts from the cell wall pores penetration and is followed by the consecutive diffusion in the intercellular space up to the root endodermis. Their migration is further hampered by Casparian strips. Subsequent transport is possible along symplastic avenues. Namely, binding to carrier proteins or aquaporins operating as water channels, endocytosis, or creation of new pores in the cell membrane [40,41]. After entering the cytoplasm, NPs interact with chloroplast, nucleus, and other structures responsible for metabolic processes at the cellular level. The following changes in metabolism alter nutrients content [42,43], plant growth [44,45], production of biomass, and finally, the plant productivity [46,47].

Photosynthesis is presumably the most important biological process which prompted plant species to inhabit the Earth. Notably, it is very sensitive to environmental variations, and therefore, photosynthesis-related parameters are among the most reliable plant stress indicators. Foundations of photosynthesis have been successfully investigated over the years, and its mechanism is quite well understood as yet [48,49,50]. However, further studies on the influence of new emerging pollutants with special emphasis put on ENPs are clearly needed.

This study continues our work on *Pisum sativum* L. as a as a model plant, cultivated worldwide, which is well suited for investigating additive interactions induced by ENPs. As pointed out by Hatami et al. [51], Tighe–Neira et al. [52] and Poddar et al. [53], the latter may either promote or hamper photosynthesis efficiency. Soilless, hydroponic cultivation, which prompts accurate plant growth control, was applied. It facilitates nutrient uptake, enables precise root separation, yields more uniform, reliable data, and finally observes the physiological changes in a more thorough way [54,55,56]. Herein, we searched for correlations between nanoceria doses and the parameters crucial for plant development, like photosynthesis indicators and the nutrients uptake by the pea plants.

## 2. Results and Discussion

### 2.1. Plant Biomass

**Figure 1:**
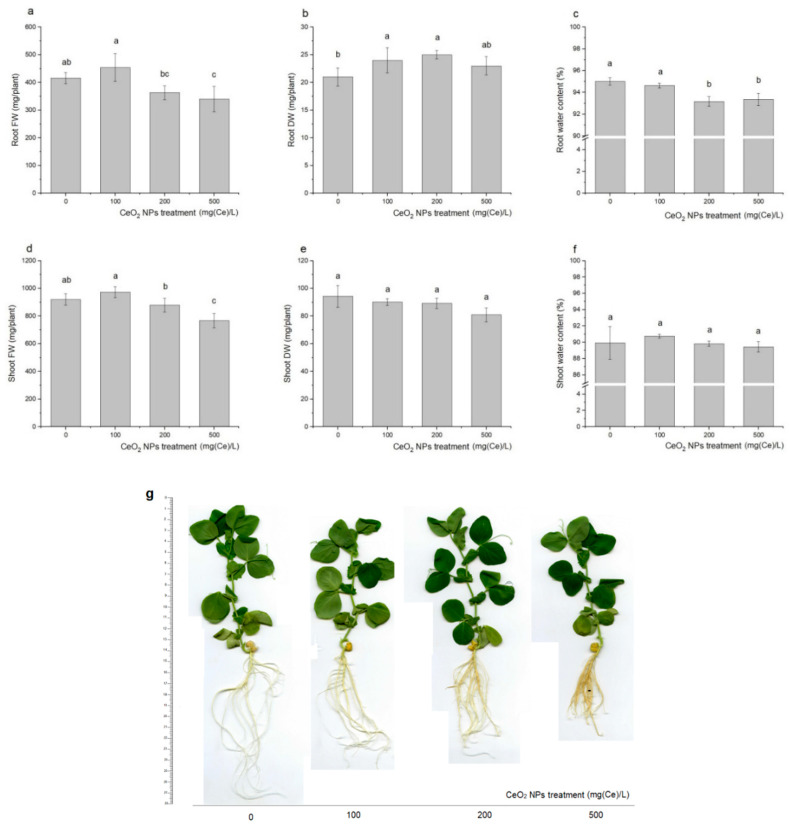
Fresh (**a**,**b**) and dry weights (**c**,**d**) augmented with the water content (**e**,**f**) in green pea plants after 12 days cultivation in Hoagland solutions supplemented with nanoparticulate CeO_2_ at the 0–500 mg/L of Ce concentrations. Data represent averages over six replicates, standard deviations are represented by vertical bars. Letters in each variable indicate statistical differences among treatments as evaluated by the Tukey’s post hoc test (α = 0.05). Roots and shoots were treated separately. Pea plant morphological changes (**g**).

The fresh and dry weights (FW and DW, respectively) are important parameters for the characterization of photosynthesis efficiency [57]. They were evaluated by the Tukey’s post hoc test at the 0.05 significance level (Figure 1). The highest impact of CeO_2_ NPs was observed for FW of either shoots or roots at the 500 mg/L concentration, while DW of shoots did not change upon any of the treatments. Following the Schwabe et al. [58] hypothesis that NPs translocation pathways are along with water flows, reduction in the water content may result from the plant’s defense strategies to mitigate NPs uptake. Moreover, aggregation of nanoparticles at the root surface can create an additional barrier for water and ion uptake [59]. NPs treatments affected the FW of shoots depending on the CeO_2_ concentration. The highest reduction (17%) as compared to the control treatment was observed at 500 mg/L level. Opposite to roots, DW, and water content in shoots were not altered by the CeO_2_ administration. Similar changes were reported by Abbas et al. [60] who presented the beneficial effect of nanoceria in concentrations up to 500 mg/L, which stimulated the wheat biomass production. Gui et al. [61] came to identical conclusions when considering the CeO_2_ NPs (50, 100, and 1000 mg/L) as a soil supplementation for lettuce cultivation. Three different responses of plants on the nanoceria supplementation were observed. The lowest concentration of NPs in the growing medium did not influence the plant growth; the moderate stimulated the plant growth with the unknown mechanism, while the highest one was toxic. The fertilizing effect on plant growth parameters in cilantro was observed by Morales et al. [62], who pointed out that root and shoots lengths strictly depended on CeO_2_ dose. They concluded that 125 mg/L was the optimal concentration. Additionally, this dose induced increasing catalase activity in shoots and ascorbate peroxidase in roots. On the contrary, Zhao et al. [63] suggested that nanoparticulate CeO_2_ soil supplementation at 400 and 800 mg/L did not affect gas exchange in leaves, the number of leaves, leaf area, shoot length, plant dry weight, and corn biomass production. However, the thorough study of Rico et al. [64] on barley cultivation has indicated that grain formation may be inhibited without visible signs of NPs toxicity on the plant. In general, divergent responses of plant species to nanoparticles are profoundly determined by genetic factors [37,65,66]. The morphological structure of roots changed substantially upon nanoparticle treatment. The decrease of roots elongation was coupled with the intensive lateral root development (Figure 1g). The latter may be supported by endogenous abscisic acid and auxin signaling [67].

### 2.2. Cerium Migration and Accumulation

Total Ce contents in roots and shoots of pea plants grown in Hoagland solution supplemented with CeO_2_ NPs are presented in Table 1. The Ce content in pea roots was in pace with its increased concentration in the growing media and suggested a passive mechanism of Ce uptake. The latter is driven by the concentration gradient across the cell membrane of roots [68]. The green pea tolerance for nanoceria, as indicated by tolerance index (TI), declined following the increasing concentrations of Ce in hydroponic solution, Table S1. The intensity of cerium uptake was described by the transfer coefficient (TC) and the bioaccumulation factor (BAF), Table S2. Nanometric CeO_2_ significantly altered either TC or BAF among all treatments and finally affected the Ce uptake. A modest saturation effect similar to that reported for soybean plants at 0. 1 g/L by Li et al. [69] was identified. The upward Ce transport was hampered as indicated by the low translocation factor (TF), which approached values, close to 0.01 (Table S1). This suggests that a concentration-independent cerium translocation with retention of Ce at the root level is the plant’s major defense strategy. Similar mechanisms are developed by plants to mitigate stress triggered by heavy metals [70,71,72]. An analogous effect has also been reported for several hydroponically grown plants, like mesquite [73], kidney bean [74], wheat, and pumpkin [58]. Similarly, White [75] pointed out that removing toxic elements from the symplastic pathway may be prompted by the sequestration in root cells vacuoles. Furthermore, the immobilization of Ce transported by apoplast in Casparian strips cannot be neglected. However, the ability of nanoparticles to cross the apoplastic barrier is also acknowledged [41,76]. In particular, Li et al. [77] revealed that in hydroponically grown tomato, the nanoparticulate CeO_2_ penetrated the interior of roots by the combination of either symplastic or apoplastic mechanisms driven by the micellar surface charge.

### 2.3. Gas Exchange

Metal homeostasis should not be investigated without the evaluation of pea plant growth and its metabolism. The latter may be examined by gas exchange parameters, namely, leaf net photosynthesis, sub-stomatal CO_2_ concentration, transpiration rate, stomatal conductance, and photosynthetic water use efficiency, Table 2. Generally, low doses of CeO_2_ stimulated plant growth and photosynthesis. In particular, the administration of CeO_2_ NPs did not affect sub-stomatal CO_2_ concentration and conductance at the α = 0.05 significance level. On the contrary, the lowest concentration of nanoceria (100 mg/L) increased leaf net photosynthesis (40%) and water use efficiency (30%) as related to the control. The remaining treatments did not inflict statistically significant changes in the two latter parameters. The transpiration rate was the highest at either 100 or 200 mg/L administrations. Notably, the 500 mg/L treatment reduced it to the value close to that recorded for the control. At moderate concentrations, electron-conducting properties of CeO_2_ NPs [78,79] accelerate the photochemical phase of photosynthesis. The increased flow of electrons boosts the quantum yield of the photosystem II and raises either NADPH or ATP production [27,80]. The latter provides energy for the Calvin-Benson cycle and enhances CO_2_ fixation as promoted by RuBisCo [52,81,82]. The improved efficiency of photosystem II in canola treated with CeO_2_ NPs was reported by Rossi et al. [83]. Cao et al. [84] pointed out that a beneficial effect of CeO_2_ was observed in soybeans at strictly defined concentrations below 500 mg/kg. Higher doses have the opposite effect. Therefore, nanoceria may be a promising photosynthesis and metabolism regulator for smart agriculture in the future. However, this complicated issue deserves further studies related to the plant species susceptibility and the thorough characterization of nanoparticles with special emphasis directed towards the toxicity evaluation [84,85,86,87].

### 2.4. Leaf Photosynthetic Pigments

**Figure 2:**
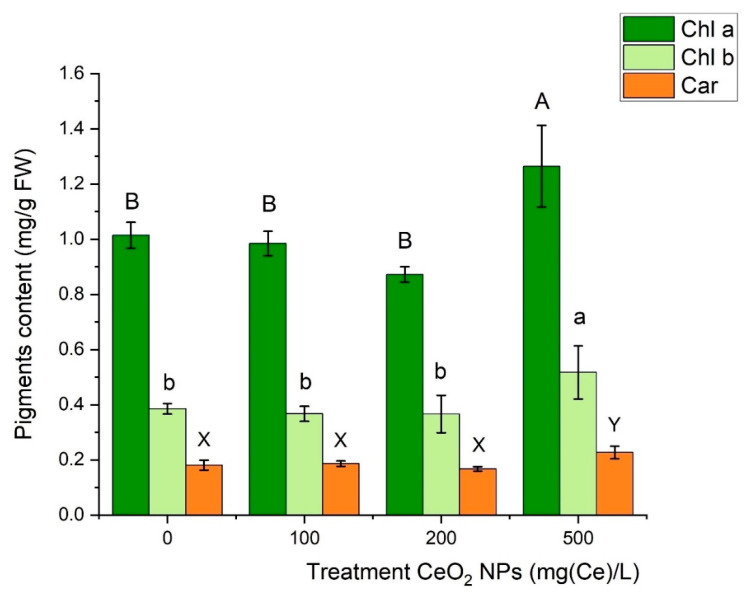
Contents of chlorophyll a (Chl a), chlorophyll b (Chl b), and carotenoids (Car) in green pea cultivated in Hoagland solutions supplemented with CeO_2_ NPs. All pigments were extracted from mature leaves. Distinct letters indicate statistically significant differences as evaluated by the Tukey’s post hoc test (α = 0.05).

Contents of chlorophyll a (a), chlorophyll b (b), and carotenoids (c) in pea cultivated in Hoagland solutions augmented with CeO_2_ NPs are presented in Figure 2. Pigments increase (25%, 34%, and by 25%, respectively) and were observed for the highest nanoceria (500 mg/L) supplementation only. Notably, Priester et al. [88] reported a decrease of photosynthetic pigments in soybean plants subjected to the CeO_2_ NPs at 100–1000 mg/kg concentrations. They pointed out that the latter effect is correlated with the ROS overproduction in leaves as determined by the fluorescent method. A decline of chlorophyll content was also reported in wheat by Du et al. [89] (400 mg/kg) and in rice by Rico et al. [90] (125 and 500 mg/kg). The opposite effect was observed by Rossi et al. [83] in soil-grown canola (200 and 1000 mg/kg) and was explained by magnesium accumulation in green parts of this plant. Unfortunately, in this study, we could hardly find a correlation between Mg and chlorophyll concentrations in leaves (Table 1). Cerium oxide NPs have the ability to enter the chloroplast [91,92]. Their oxygen vacancies, as observed in the CeO_2_ crystals, are likely to quench ROS (which are produced in chloroplast). They further increase photosynthesis efficiency and finally elevate the chlorophyll content index [93,94,95]. Thus, the ROS scavenging ability of nanoceria hampers cell destruction and furthermore mitigates environmental stress in plants [87,94,95,96,97]. In this study, the administration of CeO_2_ NPs at a high concentration of 500 mg/L distinctively reduced leaf area as compared to the control (Figure S1) and simultaneously increased carotenoids content. They act as photoprotectants by suppressing ROS and limiting oxidative damage [98,99].

### 2.5. Plant Uptake and Accumulation of Mineral Nutrients

Analysis of mutual, additive interactions between nutrients in plants is indispensable for the thorough characterization of metal homeostasis. The latter is partly visualized by the Pearson correlation coefficients matrix (Figure S3) and translocation factors (Figure S2). Administration of nanoceria had a significant effect on metal accumulation in roots hampering the uptake of Cu, Zn, Mn, Fe, and Mg, while elevating Ca content (Table 1). The latter relationship is not visible in shoots where accumulation was limited to 42% and 64% at 200 and 500 mg/L of CeO_2_ NPs, respectively. Calcium cations act as a secondary messenger that may be activated by diverse stress factors [100,101]. Therefore, it is likely that root contact with CeO_2_ NPs triggers an overproduction of ROS, stimulates Ca^2+^ ion channels, and finally, prompts an increase of calcium content in the root. A similar effect was described by Ma et al. [102], who observed that accumulation of calcium in roots of *Arabidopsis thaliana* plant was elevated upon contact with CeO_2_ NPs. On the other hand, Pošćić et al. [103] found antagonistic interactions between calcium and cerium in *Brassica napus* L. plants, which hamper active transport of Ce to the root cells. All nanoceria supplementations prompted a decrease of Fe content in pea tissues, with roots being the most affected. This constrained uptake presumably follows the down-regulation of IRT1 and IRT2 iron-transporters as induced by CeO_2_ nanoparticles [104]. Analogous processes employing IRT carriers, evolved by plants to curb the negative influence of metal-based nanoparticles, were reported by Taylor et al. [105]. Our results unequivocally show that both Cu and Mn content in pea tissues were affected by the exposure to CeO_2_ NPs. The Mn content in pea roots was decreased by Ce at concentrations of 200 and 500 mg/L, while the Zn content reduction was proportional to the Ce concentrations over the investigated range. Pearson coefficient (−1.0) indicates that at α = 0.05 significance level, an antagonism exists between the accumulation of Zn and Ce in roots. A similar effect was also detected in shoots for Mn and Fe (−0.96 and −0.95, respectively). The TF values calculated for control plants decreased along with the order Ca > Mg > Cu > Zn > Mn > Fe. The most efficiently transported of the investigated metals were macronutrients Ca and Mg and micronutrient Cu. Supplementation of Hoagland’s medium with CeO_2_ NPs changed the position of Fe in TF raw (Ca > Mg > Cu > Zn > Fe > Mn) at either 100 or 200 mg/L of Ce. The highest Ce concentration (500 mg/L) decreased TF for Ca and surprisingly prompted the transfer of all the other elements. In general, the addition of nanoceria hampered the accumulation of Cu, Zn, Mn, Fe, and Ca in pea shoots. Magnesium behaved in a less predictable way. Initially, its content in shoots decreased by 9% at 100 mg/L Ce administration. A further increase of Ce supplementation recovered Mg levels to that observed in the control sample. Magnesium cation coordinated with the porphyrin ring forms the chlorophyll active site [106,107]. It is also crucial for the activity of RuBisCO, and finally, the C3 carbon fixation pathway [108,109,110]. There are reports that Ce^3+^ can bind to the chlorophyll porphyrin ring and substitute Mg^2+^. In this way, cerium may prompt photosynthesis under the Mg deficiency [111,112,113]. Chlorophyll biosynthesis and electron transport chain are affected by the iron level [114,115,116]. The Fe concentrations in pea shoots under CeO_2_ administration (Table 1) were below the deficiency level (<100 mg/kg), as suggested by Kirby [117] in the highly respected monography *Marschner’s Mineral Nutrition of Higher Plants*. Nevertheless, no clear symptoms of the Fe deficiency, like chlorosis, were observed in any treatment. Iron, manganese, zinc, and copper are cofactors of several antioxidant enzymes (i.e., SOD, CAT, APOX), which activities may be hampered under metal-deficiency [115,118]. Cerium oxide nanoparticles affect standard pathways of plant nutrition [119,120,121,122]. However, their directions may be affected by plant species, growth media, or nutrients [102]. In soil-grown barley, nanoceria increased Fe, Cu, and Zn accumulation in leaves [64], while a decrease of the Fe content was observed in wheat [42]. Barrios et al. reported changes in nutrients accumulation in tomato plants [123] and fruits [124] exposed to CeO_2_ NPs. They highlighted the impact of cation-nanoparticle interactions on a surface coating. Nevertheless, according to Corral-Diaz et al. [125], cerium oxide nanoparticles did not affect the level of Ca, Mg, Fe, Mn, and Cu accumulation in radish roots and leaves.

## 3. Materials and Methods

### 3.1. Cerium Oxide Nanoparticles

CeO_2_ NPs were purchased from the Byk (Byk-Chemie GmbH, Wesel, Germany) as a commercially available product named Nanobyk 3810. A detailed characterization of CeO_2_ NPs has already been published by Skiba and Wolf [22]. Liquid dispersion of CeO_2_ NPs was stabilized with ammonium citrate. The average particle size as determined by transmission electron microscopy (TEM) was 25.8 ± 13.9 nm.

### 3.2. Plant Growth and Exposure

A detailed description of the applied methodology was published by Skiba and Wolf [22]. The green pea seeds, quality class A, were purchased from “PNOS” Co. Ltd., Ożarów Mazowiecki, Poland. Seeds were submerged in 70% ethanol for 10 min and then rinsed three times with distilled water. The sterilized seeds were germinated on moistened filter paper in Petri dishes in darkness for 3 days. Later, the pea seedlings were transferred to a hydroponic system filled with the aerated Hoagland nutrient solution containing: KNO_3_ (0.51 g/L), MgSO_4_·7H_2_O (0.49 g/L), CaNO_3_·4H_2_O (1.18 g/L), KH_2_PO_4_ (0.14 g/L), H_3_BO_3_ (0.6 mg/L), MnCl_2_·4H_2_O (0.4 mg/L), ZnSO_4_·7H_2_O (0.05 mg/L), CuSO_4_·5H_2_O (0.05 mg/L), Fe-EDTA (10.28 mg/L) and Na_2_MoO_4_·2H_2_O (0.02 mg/L) at pH 5.9. This medium was used without further sterilization. After four days of plant growth in raw conditions, nutrient solutions were supplemented with CeO_2_ NPs. Three concentrations: 100, 200, and 500 mg (Ce)/L were applied. Pure Hoagland medium served as the control. Fresh liquid media were provided after every 48 h intervals. The cultivation was carried out in the light of 170 µmol/m^2^ s intensity and 16/8 day/night photoperiod. Plants were collected after 12 days of CeO_2_ NPs administration at 15 BBCH scale of the growth stage.

### 3.3. Biomass Determination

At the end of cultivation, all plants were removed from the nutrient solution. The roots were carefully rinsed with demineralized water, gently dried with a filter paper, and separated from the shoots. To evaluate phytotoxicity induced by CeO_2_ NPs, fresh weights (FW) of each tissue were measured. Dry weights (DW) were determined after drying to a constant weight at 55 °C. All weights are referred to as the total root or shoot biomass calculated per single plant (mg/plant). The percentage of water contents in pea tissues was calculated using the formula of Mazaheri Tirani et al. [126].

### 3.4. Gas Exchange Parameters

Leaf net photosynthesis (A), sub-stomatal CO_2_ concentration (C_i_), transpiration (E), stomatal conductance (g_s_), and photosynthetic water use efficiency (WUE) were measured using a portable gas exchange analyzer (CIRAS-3; PP systems, Amesbury, MA) equipped with a LED Light Unit (RGBW). All those parameters were measured after 12 days of CeO_2_ administration on fully expanded leaves at the fourth node. The following settings were used: A temperature of 25 °C, reference CO_2_ concentration in chamber 390 µmol/mol, PARi 1000 µmol/m^2^s, and relative humidity 80%.

### 3.5. Photosynthetic Pigments

Chlorophyll a, b, and carotenoids contents were determined as described by Oren et al. [127]. The pigments were extracted from 200 mg of fresh leaves through grinding the tissue with 80% acetone. After incubation in a dark and cold place, the samples were centrifuged at 4000 rpm for 10 min. The absorbance of the extracts was measured at 470, 646, and 663 nm by SpectraMax i3x (Molecular Devices, San Jose, CA, USA). The pigment contents were calculated by the formula as described by Lichtenthaler and Wellburn [128].

### 3.6. Metal Content Determination in Plant Tissues

Dry roots and shoots were digested in a closed microwave system Multiwave 3000, Anton Paar. A mixture of concentrated HNO_3_ and HCl (6:1, *v*/*v*) was applied. The Cu, Zn, Mn, Fe, and Mg concentrations were determined by the High-Resolution Continuum Source Atomic Absorption spectrometer HR-CS AAS (contrAA300, Analytik Jena, Jena, Germany), while for Ca and Ce, the Inductively Coupled Plasma-Optical Emission Spectrometer ICP-OES (PlasmaQuant PQ 9000, Analytik Jena) was used.

### 3.7. Transfer Coefficient, Bioaccumulation Factor, Translocation Factor, and Tolerance Index

Transfer coefficients and bioaccumulation factors were calculated as ratios of cerium contents in roots and shoots related to cerium concentration in Hoagland solution, respectively [21,129,130]. The translocation factor was defined as the ratio of particular element content in shoots to its content in roots [131,132,133]. The tolerance index is the average root length of Ce-treated plants divided by root length measured for plants growing in reference conditions [134,135]. Root lengths were presented in Supplementary Table S1.

### 3.8. Statistical Analysis

Analyses were replicated six times per treatment, and final results are presented as an average ± SD (standard deviation). The initial hypothesis on equal variances of investigated populations were validated with the Bartlett and Hartley tests [136]. The normality of the sample distributions was subsequently proved by the Shapiro–Wilk test [137]. The one-way analysis of variance was followed by the Tukey’s post hoc test for the detailed pairwise comparison [138]. The OriginPro 2016 (OriginLab Corporation, MA, USA) was used. Pearson’s correlation coefficients between elements as determined in either roots or shoots, and the final correlation matrix were computed in the RStudio, version 1.1.463 [139]. A correlation matrix was visualized using the corrplot package [140]. The significance level was α = 0.05.

## 4. Conclusions

This work is a part of our ongoing investigations on metal migration strategies in plants induced by metal oxide nanoparticles. An increasing abundance of anthropogenic nanomaterials makes them important stressors for plants. On the other hand, several heavy metals are crucial for proper plant development. We clearly observed that nanoceria affected nutrients uptake and transport at all concentrations applied. Despite relatively high CeO_2_ administration (500 mg/L), no morphological symptoms of phytotoxicity in *Pisum sativum* L. were detected. Leaf net photosynthesis, water use efficiency, and fresh biomass production were stimulated at the 100 mg/L Ce concentration in Hoagland solution and hampered at higher levels. Presumably, this effect is initiated by catalytic properties of CeO_2_ NPs, which accelerate the photochemical phase of photosynthesis. Therefore, a change in the nutritional value of peas exposed to CeO_2_ NPs is quite likely indeed and deserves further studies. Our future investigations will focus on the dual role of nanoceria in agricultural plants. This substance was identified as a plant stressor capable of initiating production of ROS. However, at certain concentrations nanoceria also exhibit a ROS scavenging ability and support the plant’s defense mechanisms.

## Figures and Tables

**Table 1:** Contents of cerium and nutrients in roots and shoots of green pea cultivated in Hoagland solutions supplemented with nanoparticulate CeO_2_. Data are means ± SD (*n* = 6). One-way ANOVA was used to evaluate treatment effects. Statistically significant differences at α = 0.001 are indicated with ***, those at α = 0.01 are shown with **, while those at α = 0.05 are indicated with *. Letters a, b, c, and d indicate statistical differences among treatments as evaluated by the Tukey’s post hoc test (α = 0.05).

CeO_2_ NPs Treatment (mg (Ce)/L)	Metal Contents (mg/kg DW)						
	Roots						
	Ce	Cu	Zn	Mn	Fe	Ca	Mg
0	nd ^1^	12.54 ± 0.60 a	97.8 ± 4.5 a	102.1 ± 5.8 a	320 ± 19 a	4746 ± 383 c	2379 ± 74 a
100	15,216 ± 220 a	9.40 ± 0.66 bc	68.8 ± 2.7 b	101.0 ± 9.6 a	176 ± 10 b	5268 ± 188 bc	2044 ± 100 b
200	18,478 ± 1143 a	8.41 ± 0.33 c	56.6 ± 4.2 c	71.6 ± 6.4 b	161 ± 14 b	5723 ± 179 ab	1965 ± 55 b
500	26,040 ± 2901 b	10.00 ± 0.62 b	49.1 ± 2.1 d	52.8 ± 2.5 c	169 ± 7 b	5953 ± 187 a	2144 ± 100 a
ANOVA	***	***	***	***	***	**	**
	**Shoot**						
	**Ce**	**Cu**	**Zn**	**Mn**	**Fe**	**Ca**	**Mg**
0	nd	9.86 ± 0.32 a	60.5 ± 2.2 a	36.7 ± 3.8 a	105 ± 11 a	19,493 ± 512 a	3891 ± 91 a
100	101 ± 3 a	8.69 ± 0.70 b	51.7 ± 2.6 b	30.8 ± 1.9 b	93 ± 3 ab	17,564 ± 327 b	3546 ± 64 b
200	198 ± 21 b	8.38 ± 0.61 b	52.2 ± 2.0 b	26.8 ± 1.2 b	81 ± 4 b	17,165 ± 360 b	3766 ± 65 ab
500	243 ± 38 b	8.97 ± 0.62 ab	55.4 ± 0.9 b	27.3 ± 2.7 b	65 ± 8 c	14,593 ± 835 c	3873 ± 202 a
ANOVA	***	*	***	***	***	***	*

^1^ nd—not detected.

**Table 2:** Gas exchange parameters of green pea cultivated in Hoagland solutions with CeO_2_ NPs supplementation at 0–500 mg/L of Ce. Leaf net photosynthesis (A), Sub-stomatal CO_2_ concentration (Ci), Transpiration (E), Stomatal conductance (gs), and Photosynthetic water use efficiency (WUE). Data are means ± SD (*n* = 6). One-way ANOVA was used to evaluate treatment effects. Statistically significant differences at α = 0.001 are indicated with ***, those at α = 0.01 are shown with **. Letters a and b indicate statistical differences among treatments as evaluated by the Tukey’s post hoc test (α = 0.05).

CeO_2_ NPs Treatment (mg (Ce)/L)	A	Ci	E	gs	WUE
	(µmol CO_2_/m^2^s)	(µmol/mol)	(mmol H_2_O/m^2^s)	(mmol H_2_O/m^2^s)	(mmol CO_2/_mol H_2_O)
0	12.9 ± 1.6 b	293 ± 9 a	8.49 ± 0.43 b	407 ± 7 a	1.53 ± 0.24 b
100	18.1 ± 1.3 a	277 ± 14 a	10.22 ± 0.42 a	554 ± 58 a	1.98 ± 0.17 a
200	14.8 ± 1.4 b	292 ± 10 a	10.63 ± 0.07 a	548 ± 19 a	1.50 ± 0.14 b
500	14.4 ± 1.7 b	299 ± 8 a	7.73 ± 1.04 b	488 ± 156 a	1.54 ± 0.10 b
ANOVA	**	ns ^1^	***	ns	**

^1^ ns—not significant.

## Supplementary Materials

Supplementary materials can be found at https://www.mdpi.com/1422-0067/21/22/8497/s1. Table S1: Root lengths and tolerance indices (TI) of green pea plants cultivated in Hoagland solutions with CeO_2_ NPs supplementation at 0–500 mg/L of Ce. Letters a, b and c indicate statistical differences among treatments as evaluated by the Tukey’s post hoc test (α = 0.05)., Table S2: Transfer coefficient (TC), bioaccumulation factor (BAF) and translocation factor (TF) of cerium in green pea plants cultivated in Hoagland solutions with CeO_2_ NPs supplementation at 0–500 mg/L of Ce, Figure S1: Representative leaves of green pea plants cultivated in Hoagland solutions with CeO_2_ NPs supplementation at 0–500 mg/L of Ce, Figure S2: Translocation factor (TF) of macronutrients (a) and micronutrients (b) in green pea plants cultivated in Hoagland solutions with CeO_2_ NPs supplementation at 0–500 mg/L of Ce. Bars on the chart represent the value with standard deviations. Distinct letters and symbols indicate statistically significant differences as evaluated by the Tukey’s post hoc test (α = 0.05). Figure S3: Relationship between elements content in roots and shoots (a) indicated by Pearson correlation coefficients (b). Highest correlations are shown in bold (α = 0.05).

## Abbreviations

ENPs	Engineered nanoparticles
NPs	Nanoparticles
FW	Fresh weights
DW	Dry weights
TI	Tolerance index
TC	Transfer coefficient
BAF	Bioaccumulation factor
TF	Translocation factor
A	Leaf net photosynthesis
Ci	Sub-stomatal CO_2_ concentration
E	Transpiration
Gs	Stomatal conductance
WUE	Water use efficiency
Chl a	Chlorophyll a
Chl b	Chlorophyll b
Car	Carotenoids
ROS	Reactive oxygen species
PARi	Photosynthetically active radiation
