# Supplementary material for: Metal Homeostasis and Gas Exchange Dynamics in Pisum sativum L. Exposed to Cerium Oxide Nanoparticles
Source: Int J Mol Sci. 2020 Nov 11;21(22):8497. doi: 10.3390/ijms21228497 (PMC7696629; doi:10.3390/ijms21228497)
Supplement: Supplementary file 1 [file ijms-21-08497-s001.zip › Figures captions_corrected.docx]

Metal Homeostasis and Gas Exchange Dynamics in *Pisum Sativum* L. Exposed
to Cerium Oxide Nanoparticles

Elżbieta Skiba ^1,*^, Monika Pietrzak ^1^, Magdalena Gapińska ^2^ and Wojciech M. Wolf ^1^

1. Institute of General and Ecological Chemistry, Lodz University of Technology, 90-924 Lodz, Poland; elzbieta.skiba@p.lodz.pl (E.S.); monika.pietrzak@dokt.p.lodz.pl (M.P.); wojciech.wolf@p.lodz.pl (W.M.W)
2. University of Lodz, Faculty of Biology and Environmental Protection, Laboratory of Microscopic Imaging and Specialized Biological Techniques, 90-237 Lodz, Poland; magdalena.gapinska@biol.uni.lodz.pl (M.G.)

***** Correspondence: elzbieta.skiba@p.lodz.pl; Tel.: +48 42 631 31 23

**Figures captions:**

**Figure 1.** Fresh **(a,b)** and dry weights **(c,d)** augmented with the water content **(e,f)** in green pea plant after 12 days cultivation in Hoagland solutions supplemented with nanoparticulate CeO_2_ at the 0-500 mg/L of Ce concentrations. Data represent averages over six replicates, standard deviations are represented by vertical bars. Letters in each variable indicate statistical differences among treatments as evaluated by the Tukey’s post hoc test (α = 0.05). Roots and shoots were treated separately. Pea plant morphological changes **(g)**.

**Figure 2.** Contents of chlorophyll a (Chl a), chlorophyl b (Chl b), and carotenoids (Car) in green pea cultivated in Hoagland solutions supplemented with CeO_2_ NPs. All pigments were extracted from mature leaves. Distinct letters indicate statistically significant differences as evaluated by the Tukey’s post hoc test (α = 0.05).

**Supplementary Figure S1.** Representative leaves of green pea plants cultivated in Hoagland solutions with CeO_2_ NPs supplementation at 0-500 mg/L of Ce.

**Supplementary Figure S2.** Translocation factor (TF) of macronutrients **(a)** and micronutrients **(b)** in green pea plants cultivated in Hoagland solutions with CeO_2_ NPs supplementation at 0-500 mg/L of Ce. Bars on the chart represent value with standard deviations. Distinct letters and symbols indicate statistically significant differences as evaluated by the Tukey’s post hoc test (α = 0.05).

**Supplementary Figure S3.** Relationships between elements content in roots and shoots **(a)** indicated by Pearson correlation coefficients **(b)**. Highest correlations are shown in bold (*α* = 0.05).
