# Supplementary figures and images for: Metal Homeostasis and Gas Exchange Dynamics in Pisum sativum L. Exposed to Cerium Oxide Nanoparticles
Source: Int J Mol Sci. 2020 Nov 11;21(22):8497. doi: 10.3390/ijms21228497 (PMC7696629; doi:10.3390/ijms21228497)

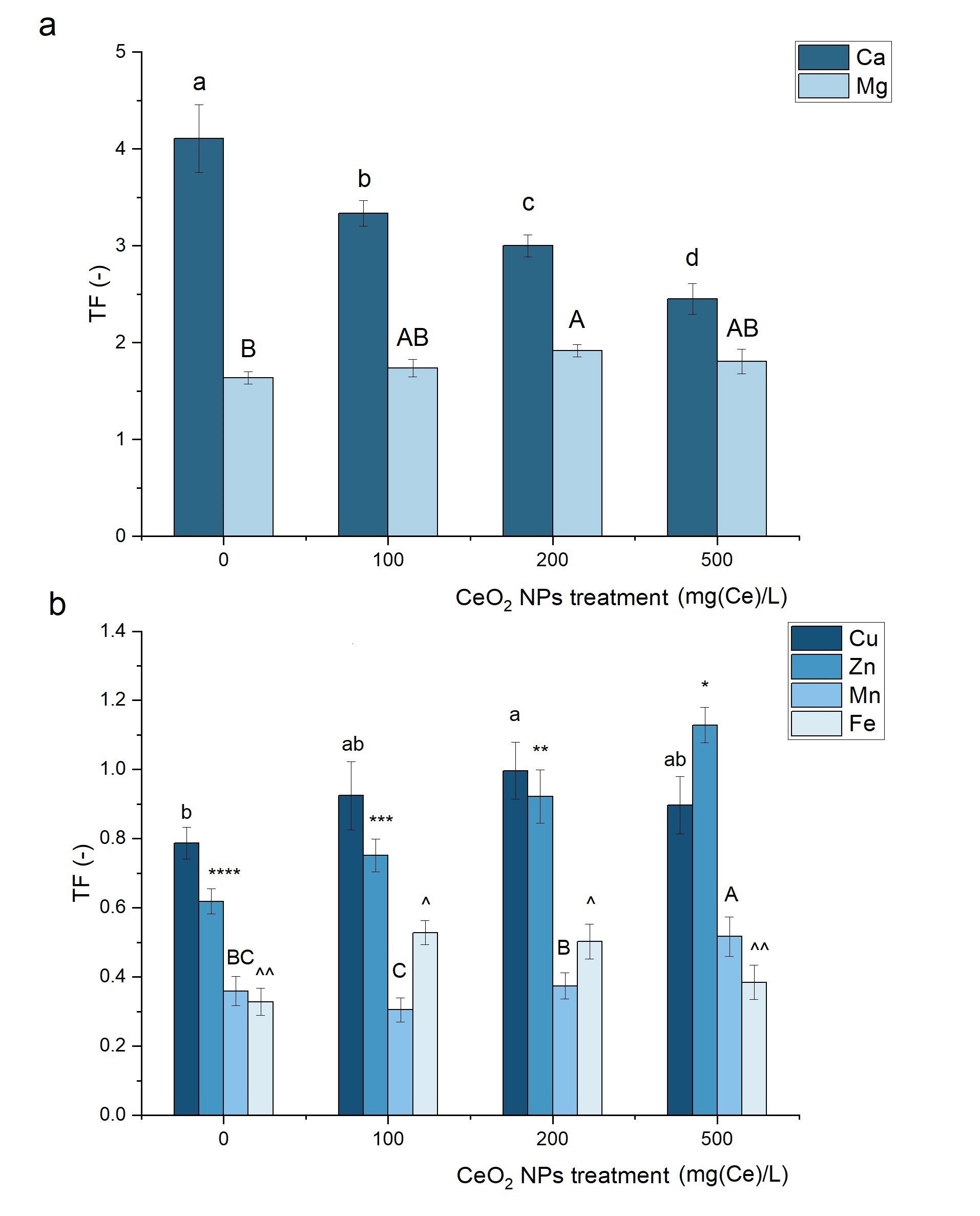

Supplement: Supplementary file 1 [file ijms-21-08497-s001.zip › Figure S2 corrected.jpg]

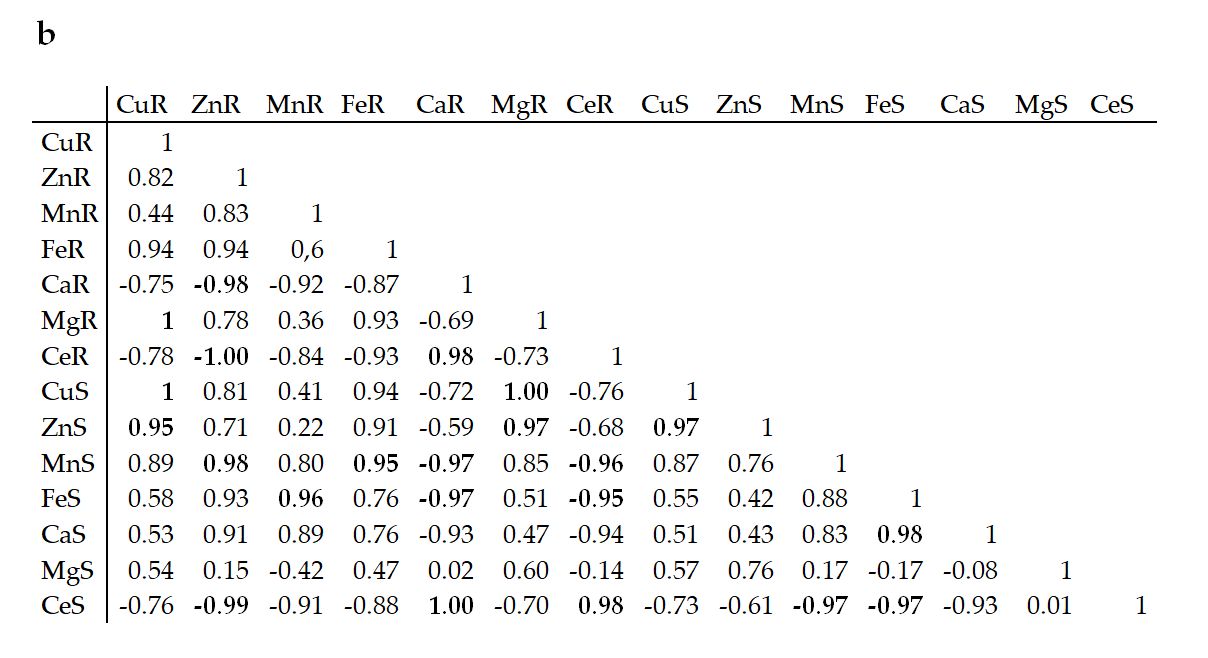

Supplement: Supplementary file 1 [file ijms-21-08497-s001.zip › Figure S3b corrected.jpg]

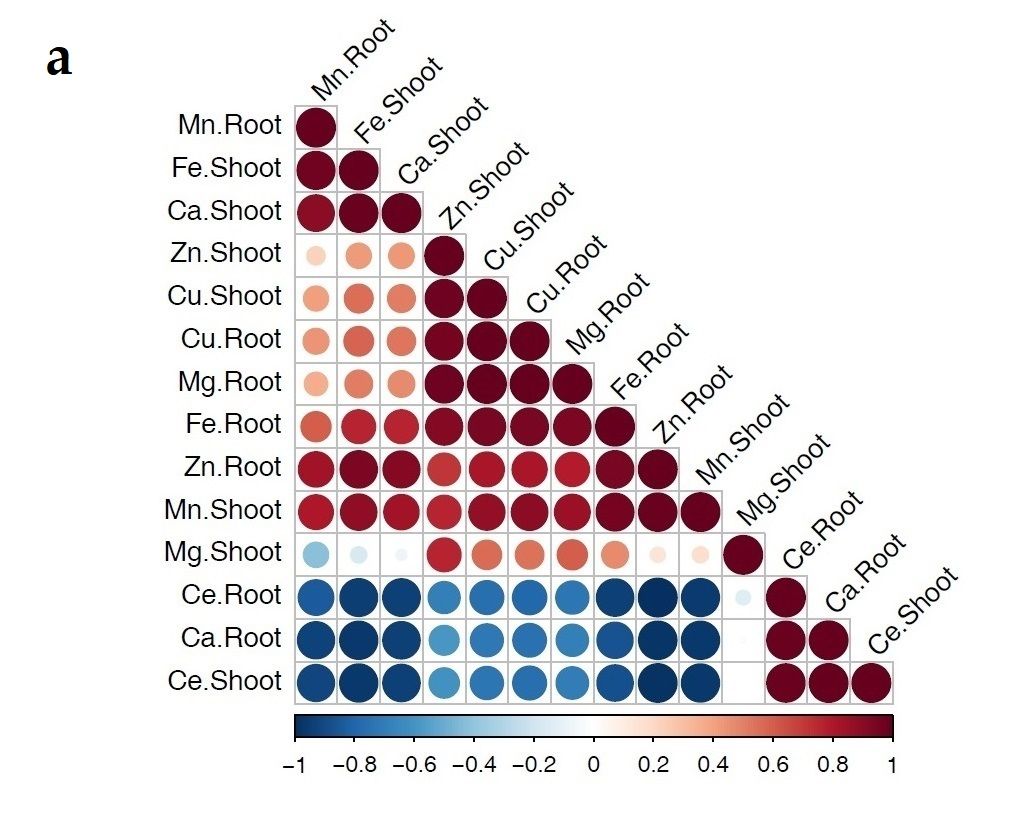

Supplement: Supplementary file 1 [file ijms-21-08497-s001.zip › Figure_S3_a.jpg]

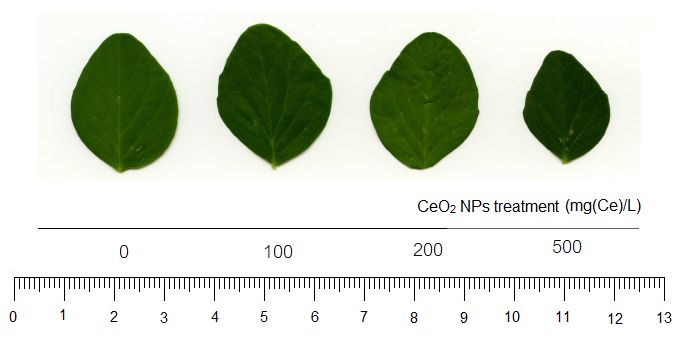

Supplement: Supplementary file 1 [file ijms-21-08497-s001.zip › Figure_S1.jpg]
